# Supplementary material for: Atomic-state diagnostics and optimization in cold-atom experiments
Source: Sci Rep. 2018 Feb 12;8:2805. doi: 10.1038/s41598-018-20522-x (PMC5809594; doi:10.1038/s41598-018-20522-x)
Supplement: Supplementary file 1 — Supplementary Information [file 41598_2018_20522_MOESM1_ESM.pdf]

Atomic-state diagnostics and optimization in cold-atom experiments

Krystian Sycz, Adam M. Wojciechowski, Wojciech Gawlik

Supplemental information

Experimental arrangement

The experiments were conducted using cold rubidium atoms released from a magneto-optical trap. Two-cell-system prepared 10^8 to 10^9 cold atoms of either ^{85}Rb or ^{87}Rb (for reference see Table 1) in a volume of a few mm^3 at about 100 μK in ultra-high vacuum (initial vacuum pressure below 10^{-10} mbar).

The cooling and trapping light for both the 2D and 3D MOT, as well as for the pushing beam, was delivered from a commercial tapered amplifier laser system. A home-built diode laser provided the repumping light. An independent external-cavity diode laser was used for measurements of magneto-optical rotation and atomic state preparation. Its frequency was stabilized with either a saturated absorption or a Doppler-Free Dichroic Laser Lock (DFDL)[1] spectroscopy setup. Light from this laser is split into two beams, the pump and the probe, both about 1.5 mm in diameter at the atomic cloud volume. Their intensities and detunings are independently controlled by acousto-optical modulators (AOMs), each being in a double-pass configuration. Both laser beams can therefore be precisely tuned around the desired transition of either of the rubidium isotopes. Results presented in this work refer to the rubidium D_2 line cyclic transitions: F'=4 – F=3 for ^{85}Rb (Figures: 1-3a, 4a-b, 6, S1-S5) and F'=3 – F=2 for ^{87}Rb (Figures: 3b, 4c-d, 5).

Care was taken to precisely compensate any transverse fields and possibly shield the system from stray fields (AC and DC). The science chamber is isolated from external magnetic fields by a cylindrical, single-layer μ-metal shielding of 10 cm in both length and diameter, and a thickness of 0.5 mm. Optical access for laser beams and imaging optics, as well as opening for the vacuum apparatus is provided by means of protruding μ-metal sleeves, about 3 times longer than the diameter of the respective opening. The shield provides a DC shielding factor of about 20. Inside the shielding, well-controlled magnetic fields are generated by six pairs of coils which create uniform and gradient magnetic fields. Home-built current sources control the magnetic field with precision better than 1 μG and perform switching of both AC and DC fields or their superposition within about 0.1 ms. To increase the long-term current stability, currents are not switched off but directed to sink circuits.

A high-quality crystal polarizer linearly polarizes the probe beam just before it enters the science cell. After passing the sample, beam polarization is analysed with a balanced polarimeter, consisting of a Wollaston prism and two avalanche photodiodes operating in a linear intensity-response mode. Simultaneous recording of both photocurrents allows us to measure the optical-rotation angle. Alternatively, the forward scattering geometry may be used with only one detection channel with a polarizer orthogonal to the linear polarizer[2]. The pump beam is crossing the sample either at a small (<2 deg) or at right angle with respect to the probe beam (along 0*x*). Its polarization is set to either linear or circular. Both beams propagate at a small angle with respect to the normal of the science cell walls in order to avoid interference effects from the reflected beams (Fig. 1b of the *main article*).

Single-beam and pump-probe configuration

Two classes of measurements were performed: one with a single beam that simultaneously created and probed given atomic states and second, the pump-probe measurement, where a weak probe interrogated the state prepared earlier by a pump beam. Depending on the polarization of the pump beam and direction of the magnetic field, in the two-beam, pump-probe measurement we observed the time-dependent free induction decay signals associated with either atomic polarization or alignment (coherences with $|\Delta m|=2$).

Measurement sequence

Timing sequence of the experiment (Fig. 1c of the *main article*) starts with loading of the main trap from the 2D MOT and through recapturing the expanding atom cloud from a previous measurement. After the loading time, the trapping optical and quadrupole magnetic fields are turned off while the desired homogenous magnetic fields are turned on. From this moment, the atomic cloud starts to expand and fall freely, while the actual rotation measurements are delayed by 5 ms to allow for the eddy currents (induced mostly by the switching of the MOT quadrupole field) to decay and the applied fields to stabilize.

In the single-beam experiments, one linearly-polarized, near-resonant beam parallel to the magnetic field oriented along the z-axis performs the readout and manipulation of the atomic state. After switching on the beam, it's polarization rotation is recorded by the polarimeter. Such experiments are referred to as measurements of the NFE.

In two-beam experiments, atoms are initially prepared in the desired state by a relatively short (<15 μs) and intense (~1 mW) pumping pulse of appropriate polarization. The circularly (linearly) polarized pump pulse, sufficiently strong (typically 1 mW/mm^2) and short when compared to Larmor frequency in a given magnetic field, creates atomic polarization (coherence) in the sample. Subsequently, a weak (below 1 μW) probe beam is turned on and its polarization rotation is recorded. The probe may be applied with appropriate delay to enable measurements of the time evolution of the rotation signals for up to some 20 ms (limit imposed by the freefall of atoms). The rotation measurement is followed by switching back the MOT fields. The expanding cloud is then recaptured and the whole sequence is repeated. The typical cycle time lasts a fraction of a second.

NFE signal buildup, amplitude and width

The characteristics of the NFE resonance strongly depend on the light intensity, as depicted in Fig. S1. Increasing light power causes the resonance to appear larger and faster. The bottom panel illustrates the broadening effect of a resonant probe light at larger intensities. The linear extrapolation of the low-intensity dependence to the zero light power (Fig. S1c) yields about 4.5 mG intercept which corresponds to about 150 μs coherence time. This is significantly shorter than expected from just the free-fall contribution ($\gamma_{TOF} \sim \frac{1}{10\text{ ms}}$), suggesting presence of other decoherence mechanisms. To study them systematically, the light-dependent contribution needs to be minimized. This is not trivial as a certain pump light intensity is necessary for the signal amplitude build-up, particularly in the one beam setup when the probing cannot be separated from pumping.

Effect of magnetic shielding on FID signals

In Fig. S2 we demonstrate the effect of the magnetic field inhomogeneity on the recorded FID signals of atomic polarization. If the compensation of stray fields is limited to the DC-field components only, the resulting FID curve is quickly damped (within about 200 μs, blue trace). Compensation of the first-order magnetic-field gradients extends the duration of the FID signal about 3 times (red signal). The former and latter FID signals have clearly non-exponential envelopes and/or exhibit some beating which indicate complexity of the relaxation processes. On the other hand, shielding of the science chamber yields regular FID curves (black trace) characterized by lower damping rate of about 1 ms for CW 70 MHz blue detuned probe of 1 $\mu W/mm^2$ intensity.

Stroboscopic measurement of the coherence FID

Coherence FID signals have the largest amplitude for probe light close to the resonance (Fig. 4c of the *main paper*). Under such conditions the FID signal decays quickly due to the decoherence caused by interaction with the probe beam. Its influence can be mitigated by replacing continuous wave probing with a pulsed scheme, specifically, by the, so called, *relaxation in the dark* approach[3]. In Figure S3 we present a coherence FID signal recorded by delaying the probe pulse relative the pump one and repeating the measurement over many realizations with several increasing delays. Although individual FID curves (shown with different colors) decay rapidly, the envelope of their maximal amplitudes corresponding to different delays and marked with dashed red lines decays slower. This is due to the fact that within the delay time between pumping and probing, the probe beam does not perturb the atoms (their relaxation occurs in the dark). Consequently, the pulsed (strobed) probing significantly slows down the decoherence, and enables observations of coherence FID signals with the decay times comparable to those seen with population signals.

Magnetic transients due to the trap switching without the shielding

Instability of both: the magnitude and the orientation of magnetic field may lead to a complex magneto-optical response. Magnetic rotation signals recorded after switching off the MOT quadrupole field and with the unshielded science chamber are visualized in Fig. S4a. The residual magnetic field results from imperfect compensation and inhomogeneity of the laboratory DC field, residual 50 Hz mains noise and decaying eddy-current-induced transients. To slow down the decoherence caused by the probe beam its frequency was blue detuned by 70 MHz. The inset to Fig. S4a presents time-dependent rotation response measured without pumping pulses, i.e. exclusively by the probe beam after switching off the MOT fields at $t = 0$. The black and red traces correspond to triggering the measurement sequence by the mains line with the 0 and 180 deg phase (the rising and falling slopes, respectively). These signals reflect mainly the effect of the longitudinal component, $\theta(B_z) \propto B_z$ of the stray field. The main part of Fig. S4a presents rotation signals acquired with additional resonant, circularly polarized pump pulses repeated every 0.7 ms, for the same triggering conditions as used for the signals in the inset. The rotation signals observed with pump pulses consist of the contributions caused by the longitudinal magnetic-field component B_z of the stray field superimposed with new oscillating contributions following each pump pulse. These contributions are the FID signals due to total magnetic field experienced by atoms at various instants. By measuring the distance between local rotation signal extrema, it is possible to estimate the Larmor precession frequency and, therefore, to read out the full magnitude of the field, $|B|$, even

without calibration of the rotation signal (Fig. S4b). The accuracy of such a procedure depends on a number of visible oscillations and a degree of FID signal distortion.

Residual magnetic instability with the shielding

The single-beam NFE rotation may also be used for diagnostics of small field transients, e.g., associated with switching off the MOT quadrupole field and applying the Faraday field. Figure S5 presents the close up of rotation signal shown in the main text (Fig. 2b), i.e., the contour plot of the rotation signal recorded in the shielded experiment as a function of time and a near-zero longitudinal magnetic field. The asymmetry and drift of rotation signal around zero applied-field reflects transient fields caused by eddy currents arising within the μ-metal shielding, as well as the onset of the applied magnetic field. The initial field instability is smaller than in the unshielded case (Fig. S4), as the probing starts 4 ms after switching off the MOT.

Table 1. A reference list for the isotopes that were used for each of the presented measurements.

Figure	1a	2a-d	3a	3b	4a,b	4c,d	5a,b	6a-c	S1-5
Isotope	^{85}Rb	^{85}Rb	^{85}Rb	^{87}Rb	^{85}Rb	^{87}Rb	^{87}Rb	^{85}Rb	^{85}Rb

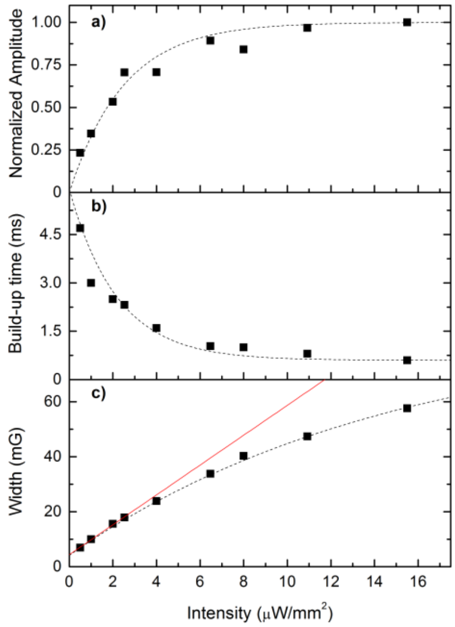

Fig. S1. Dependence of (a) amplitude, (b) width and (c) buildup time of the maximum amplitude of the NFE resonance on the resonant light intensity illustrating saturation and power broadening of the rotation resonance.

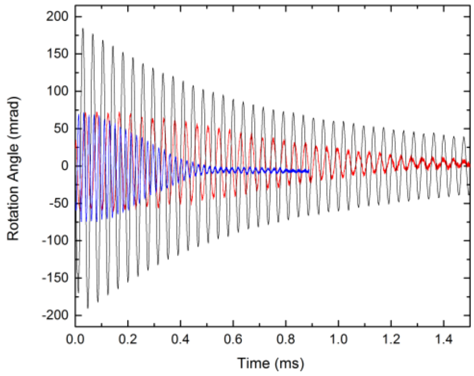

Fig. S2. FID signals of atomic polarization $\theta(t)$ recorded by a CW probe with: compensation of DC stray magnetic fields (blue); its DC value and first-order gradients (red); and with no additional compensation but with magnetic shielding of the science cell (black). B_z = 0, and B_x = 56 mG. Note the change in FID signal shape for the unshielded cases

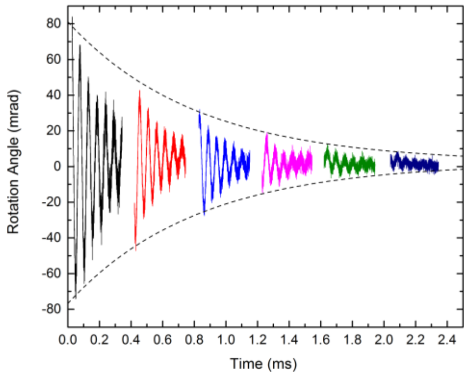

Fig. S3. Stroboscopic measurement of the coherence FID. The plot depicts many recordings of the FID oscillations with different delays between the pump and probe pulses (marked with different colors). Although individual FID curves decay rapidly because of the probe-induced decoherence, the envelope of their maximal amplitudes corresponding to different delays decays slower: typical decay time of individual FID oscillations are 170 μs, while that of the envelope of the strobed FID amplitude is 815 μs as shown by dashed lines.

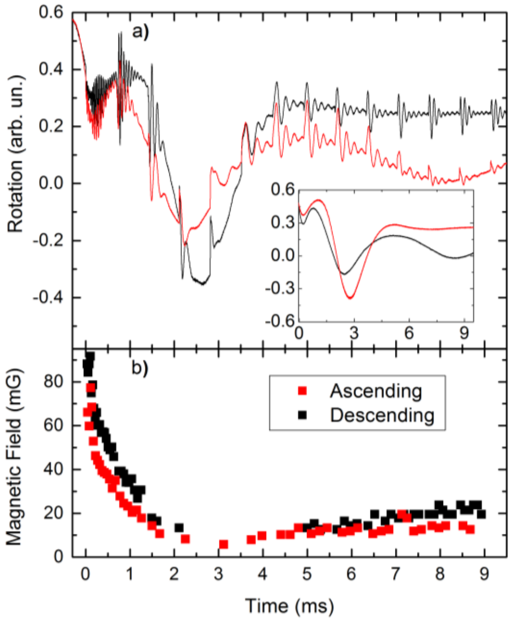

Fig. S4. Rotation signal $\theta(t)$ of a CW probe beam recorded after switching off the MOT at $t = 0$ with an unshielded MOT. (a) Two sets of signals are presented: without pumping (inset) and with periodic pumping (main panel). The red and black traces correspond to triggering by the 0 and 180 deg phases of the mains line, respectively. The signal in the inset represents the slowly-varying part in the main frame and reflects the instantaneous value of the longitudinal component of the field vector. (b) The FID oscillations following the pump pulses occur at Larmor frequency and provide a measure of the amplitude $|B|$ of the total magnetic-field. The probe was blue detuned by 70 MHz.

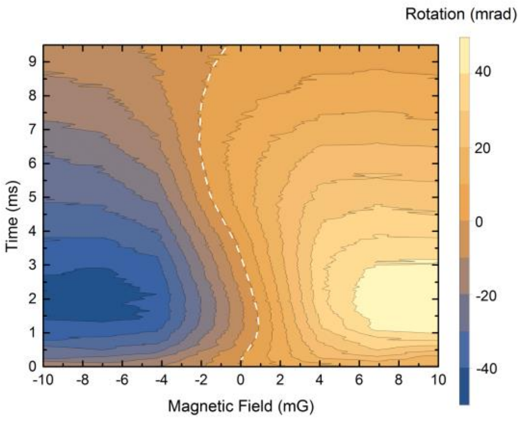

Fig. S5. Zoom into rotation signals from Fig. 2b (of the *main paper*) showing in-detail the small drifts of the resonance centre caused by switching from MOT quadrupole field to the uniform Faraday field. White broken line indicates the position of zero rotation value at various times. Signals were recorded with the magnetic shielding.

---

1 Wąsik, G., Gawlik, W., Zachorowski, J., Zawadzki, W. Laser frequency stabilization by Doppler-free magnetic dichroism. Appl. Phys. B **75**, 613-619 (2002)

2 Corney, A., Kibble, B. P., Series, G.W. The forward scattering of resonance radiation, with special reference to double resonance and level-crossing experiments. Proc. R. Soc. Lond. A, **293**, 70-93 (1966)

3 Franzen, W. Spin Relaxation of Optically Aligned Rubidium Vapor. Phys. Rev. **115**, 850-856 (1959)
